# Surface antigens contribute differently to the pathophysiological features in serotype K1 and K2 *Klebsiella pneumoniae* strains isolated from liver abscesses

**DOI:** 10.1186/s13099-016-0085-5

**Published:** 2016-02-13

**Authors:** Kuo-Ming Yeh, Sheng-Kung Chiu, Chii-Lan Lin, Li-Yueh Huang, Yu-Kuo Tsai, Jen-Chang Chang, Jung-Chung Lin, Feng-Yee Chang, Leung-Kei Siu

**Affiliations:** Department of Internal Medicine, Division of Infectious Diseases and Tropical Medicine, Tri-Service General Hospital, National Defense Medical Center, No. 325, Sec. 2, Cheng-Kung Road, Neihu, 114 Taipei City Taiwan; Infection Control Office, Tri-Service General Hospital, National Defense Medical Center, No. 325, Sec. 2, Cheng-Kung Road, Neihu, 114 Taipei City Taiwan; School of Respiratory Therapy, College of Medicine, Taipei Medical University, Taipei, Taiwan; Department of Internal Medicine, Division of Pulmonary Medicine, Shuang Ho Hospital, Taipei Medical University, New Taipei City, Taiwan; Institute of Infectious Diseases and Vaccine Research, National Health Research Institutes, 35 Keyan Road, Zhunan, 35053 Miaoli, Taiwan; Graduate Institute of Basic Medical Science, China Medical University, Taichung, Taiwan

## Abstract

**Background:**

The virulence role of surface antigens in a single serotype of *Klebsiella pneumoniae* strain have been studied, but little is known about whether their contribution will vary with serotype.

**Method:**

To investigate the role of K and O antigen in hyper-virulent strains, we constructed O and K antigen deficient mutants from serotype K1 STL43 and K2 TSGH strains from patients with liver abscess, and characterized their virulence in according to the abscess formation and resistance to neutrophil phagocytosis, serum, and bacterial clearance in liver.

**Results:**

Both of K1 and K2-antigen mutants lost their wildtype resistance to neutrophil phagocytosis and hepatic clearance, and failed to cause abscess formation. K2-antigen mutant became serum susceptible while K1-antigen mutant maintained its resistance to serum killing. The amount of glucuronic acid, indicating the amount of capsular polysaccharide (CPS, K antigen), was inversed proportional to the rate of phagocytosis. O-antigen mutant of serotype K1 strains had significantly more amount of CPS, and more resistant to neutrophil phagocytosis than its wildtype counterpart. O-antigen mutants of serotype K1 and K2 strains lost their wildtype serum resistance, and kept resistant to neutrophil phagocytosis. While both mutants lacked the same O1 antigen, O-antigen mutant of serotype K1 became susceptible to liver clearance and cause mild abscess formation, but its serotype K2 counterpart maintained these wildtype virulence.

**Conclusion:**

We conclude that the contribution of surface antigens to virulence of *K. pneumoniae* strains varies with serotypes.

## Background

*Klebsiella pneumoniae* is a common gram-negative pathogen causing both community and nosocomial infections [1]. In the past two decades, a new type of invasive *K. pneumoniae* disease has emerged in Taiwan and worldwide that typically presents as a community-acquired primary liver abscess [2, 3]. Serotype K1 and K2 strains caused more than two-third of *K. pneumoniae* liver abscess [4].

Two surface carbohydrate structures of *K. pneumoniae*, capsular polysaccharide (CPS, K antigen) and O antigen portion of its lipopolysaccharide (LPS), are important pathogenic factors [1]. As the outermost components of the bacterial surface, these structures are among the first to be encountered by the innate immune system of host.

The K and O antigens of *K. pneumoniae* represent two families of polysaccharides comprised of repeating subunits, with latter linked to the core antigen of LPS. The structure of K1 antigen is →4)-[2,3-(S)-pyruvate]-β-_D_-Glc*p*A-(1→4)-α-_L_-Fuc*p*-(1→3)-β-_D_-Glc*p*(1→, and K2 is →4)-[1,3-α-GlcUA]-β-Man-(1→4)- α-Glc-(1→3)-β- -Glc(1→ [5, 6]. O1 antigen is made of two disaccharide, d-galactan I [→3-β-Gal*f*-(1→3)-α-Gal*p*-(1→] and d-galactan II [→3-β-Gal*p*-(1→3)-α-Gal*p*-(1→] [7]. The subunits of K and O antigens were assembled separately into chains within the bacterial cytosol and subsequently transported to the outer membrane [8, 9]. The synthetic enzymes and transport mechanism of K and O antigens are encoded in by the *cps* and *rfb* gene cluster, respectively [10, 11]. Strong associations exist between the 77 K serotypes and 9 O serotypes. For instance, K1 and K2 almost always coexist with O1 [12].

The virulence role of surface antigens in a single serotype of *K. pneumoniae* strain have been studied, but little is known about whether their contribution will vary with serotype. To investigate the impact of serotype on the role of K and O antigens in the pathogenesis of *K. pneumoniae* liver abscess, we used insertion mutagenesis to construct mutants deficient of K or O antigen in serotype K1 and K2 strains and examined their ability to cause abscess formation and resist host defenses, including neutrophil phagocytosis, serum killing and liver clearance.

## Methods

### Bacterial isolates and plasmids

Two wildtype *K. pneumoniae* strains, STL43 (O1:K1) [13] and TSGH69 (O1:K2), were isolated from patients with liver abscess in Taiwan. The bacterial strains and plasmids used in this study are listed in Table 1. For general use, bacteria were routinely incubated in Luria–Bertani (LB) broth or agar at 37 °C. Kanamycin (50 μg/ml) was added to the media for selection. BIND (brilliant green containing inositol-nitrate-deoxycholate) agar was used to select *K. pneumoniae* mutants [14].

**Table 1:** Bacterial strains and plasmid used in this study

Strain or plasmid	Relevant characteristics	Reference or source
*Klebsiella pneumoniae*		
STL43	Wild type (O1:K1)	Yeh et al. [13]
STL43Δ*wzy*	K-deficient mutant of STL43	Yeh et al. [13]
STL43Δ*wbbO*	O-deficient mutant of STL43	Yeh et al. [13]
TSGH69	Wild type (O1:K2)	This study
TSGH69Δ*wzy*	K-deficient mutant of TSGH69	This study
TSGH69Δ*wbbO*	O-deficient mutant of TSGH69	This study
VGH825	Wild type (O1:K32)	This study
*Escherichia coli*		
*E. coli* S17-1λpir	Conjugation donor	Biomedal
Plasmid		
pUT-kmy	Suicide vector for insertional mutagenesis	Yeh et al. [13]

### Construction of mutants

A newly constructed plasmid, pUT-kmy [13], and primer pairs designed for mutagenesis and genotype confirmation shown in Table 2 were used for insertional mutagenesis by means of an established method [13]. With the wildtype chromosome as a template, the polymerase chain reaction (PCR)–amplified fragments of target gene were excised by *Eco*RI and *Not*I and then were ligated into the *Not*I-*Eco*RI site of pUT-kmy. The resultant plasmid constructs were electroporated into *Escherichia coli* S17–1l pir, followed by conjugation with the wildtype strain. Transconjugations were selected using BIND supplemented with 50 mg/ml kanamycin. The colony grown in BIND with kanamycin was the *K. pneumoniae* strain that had the kanamycin-resistant pUT-kmy inserted into the target gene. The mutant genotype was confirmed by PCR performed with one primer pair (known as “PUT-F3A” and “PUT-R1”) designed outside the *Not*I-*Eco*RI restriction sites of pUT-kmy and one pair (known as “ORF-OF” and “ORF-OR”) designed outside the target gene. The PCR results achieved with the use of primer pairs PUT-F3A and ORF-OF or PUT-R1 and ORFOR were positive for the target gene mutant but negative for the wildtype.

**Table 2:** List of primers in this study

Primer	Sequence	Purpose
PUT-F3A	5′-CAGGAGTACGGATAAAATGC-3′	Confirmation of genotype after insertion mutagenesis
PUT-R1	5′-AAGGTTTAACGGTTGTGGAC-3′	
K2-wzy-IF	5′-ATTTTCCAGAGTTAGACCCG-3′	Construction of TSGH69Δ*wzy*
K2-wzy-IR	5′-TGTCGTTTTGGGATTTGTAA-3′	
K2-wzy-OF	5′-GCCTTTTCATTTATACAGGA-3′	Confirmation of TSGH69Δ*wzy* genotype
K2-wzy-OR	5′-GGAATTGAAATCAACTACAG-3′	
K2-wbbO-IF	5′-GGAATTCCTGTTTGATTGGTGGTGTGCT-3′	Construction of TSGH69Δ*wbbO*
K2-wbbO-IR	5′ATAAGAATGCGGCCGCGACGGCAAAGCAACGATATT-3′	
K2-wbbO-OF	5′-AGATTCACATCAGCCATTTT-3′	Confirmation of TSGH69Δ*wbbO* genotype
K2-wbbO-OR	5′-GGAATTCCTGAGAAAATTGTGTTATTTCA-3′	

### Silver staining and western blotting

Cell surface polysaccharides, composed of CPS and LPS, were extracted by a modified hot water/phenol method [6]. In brief, bacterial colonies grown overnight in LB agar were collected, and suspended in 400 µl distilled water. Equal volume of hot phenol (65 °C, pH 6.6) was added followed by vigorous shaking at 65 °C for 20 min. Suspension were then cooled on ice, and centrifuged at 8500×*g* for 20 min. Supernatants were transferred to a centrifuge tube. Equal volume of chloroform were added, and centrifuged at 8500×*g* for 20 min. Supernatants were transferred to a centrifuge tube for further tests.

Samples were analyzed with 8 % sodium dodecyl sulfate polyacrylamide gel electrophoresis (SDS–PAGE), followed by silver staining [15], and western immunoblotting. The gel was transferred to PVDF (polyvinylidene difluoride) membrane with Trans-Blot SD Semi-Dry Electrophoretic Transfer Cell (BIO-RAD, California, US), and western immunoblotting was performed using 1:500 *Klebsiella* antisera SEIKEN (Denka Seiken, Tokyo, Japan), and 1:1000 anti-rabbit IgG (whole molecule) peroxidase (Sigma, Missouri, US). The membrane was stained with SIGMAFAST DAB with a Metal Enhancer Tablet Set (Sigma, Missouri, US).

### Quantification of CPS

The bacterial CPS was extracted with the method previous described [16]. Samples (500 μl) of overnight grown bacteria in BHI was mixed with 100 μl of 1 % Zwittergent 3-14 in 100 mM citric acid (pH 2.0). After 20 min at 50 °C, the mixture was pelleted by centrifugation, and 250 μl of the supernatant was transferred to a new tube, and 1 ml of absolute ethanol was added. The pellet was dried and dissolved in 200 μl of HCl, and then 1200 μl of 12.5 mM borax in H_2_SO_4_ was added. The mixture was vigorously mixed, boiled for 5 min, and cooled, and then 20 μl of 0.15 % 3-hydroxydiphenol was added. The glucuronic acid content was measured with the absorbance at 520 nm, and was determined from a standard curve. At least three successive tests were conducted to measure the mean content for each isolate.

### Human neutrophil phagocytosis assay

The collection and storage of human blood was approved by the Institutional Review Board, Tri-Service General Hospital, National Defense Medical Center (TSGHIRB B-102-13). The neutrophil isolation from healthy volunteers and the bacterial labeling with fluorescein isothiocyanate (FITC) were performed as previously described. The mixture of the labeled bacteria, the neutrophil suspension, the pooled human serum, and the phosphate-buffered saline (PBS; pH 7.4) was incubated for 1, 5, 15, 30 min in a shaking 37 °C water bath. Ethidium bromide was added before measurement to suppress the extracellular fluorescence. The FITC fluorescence was detected with FACScan (Becton–Dickinson Immunocytometry Systems, California, US). The mean percentage of neutrophils that carried FITC-stained bacteria in at least six successive results was designated as the phagocytosis rate.

### Serum bactericidal assay

The bacterial susceptibility to human serum was analyzed by means of an established method [17]. Early-log-phase bacteria were diluted to about 4 × 10^6^ CFU (colony-forming unit)/ml in PBS. Mixtures of 250 μl of bacterial suspensions and 750 μl of normal human serum were incubated at 37 °C. Samples were taken after incubation for 0, 1, 2 and 3 h, and serial dilutions were plated on LB agar for colony counts. At least two successive tests were done for each isolate.

### Hepatic bacterial clearance study

Mice used in this study was approved by animal used committee with NHRI-IACUC-103014-A. At least four C57BL/6 male mice aged 6 weeks were infected with sub-lethal dose (0.1 × LD_50_, lethal dose 50 %) of *K. pneumoniae* strains by an intraperitoneal route. The mice were sacrificed at 0, 3 h, and 1, 2, 3 days postinfection. Livers were aseptically removed, weighed, and homogenized separately with sterile PBS. A 100 μl aliquot from each homogenate were serially diluted tenfold with PBS and plated on LB agar. Bacterial colonies were counted after overnight incubation at 37 °C.

### Histology

Infected mice were sacrificed 3 h or 3 days postinfection. Liver was immersed in 10 % formalin, embedded, micro sectioned, mounted on microscopic slides, and stained with hematoxylin and eosin stain. The slides were microscopically observed (200×).

### Statistical analysis

Statistical analyses were performed using SPSS software package (version 17.0, Chicago, IL, USA). Analysis was performed by the Chi square test or Fisher’s exact test for categorical variables. A value of *P* < .05 was considered statistically significant.

## Results

### Construction and confirmation of mutants deficient of K or O antigen

Two wildtype *K. pneumoniae* strains causing liver abscess, STL43 (O1:K1) and TSGH69 (O1:K2), were study strains ready for mutagenesis. Two K deficient mutants, STL43Δ*wzy* and TSGH69Δ*wzy,* was constructed by the insertional recombination of *wzy*, previously known as *magA* in serotype K1 and *orf10* in K2. And our previous study showed that the complemented mutant restored its wildtype resistance to phagocytosis [13]. In correspondence with previous study to construct O deficient mutants [18], STL43Δ*wbbO* and TSGH69Δ*wbbO*, was obtained by inactivation of WbbO, a galactosyltransferase essential for synthesis of O1 antigen.

**Fig. 1:**
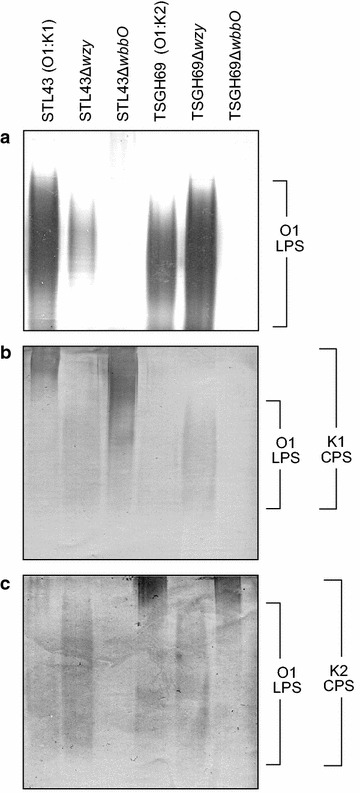
Silver staining and western blotting of surface polysaccharides extracted from six *Klebsiella pneumoniae* strains. **a** Silver staining. Western blot analysis performed using K1 (**b**) or K2 (**c**) antiserum. *CPS* capsular polysaccharide, *LPS* lipopolysaccharide, *STL43* serotype O1:K1 wild type, *STL43Δwzy* K-antigen-deficient mutant, *STL43ΔwbbO* O-antigen-deficient mutant, *TSGH69* serotype O1:K2 wild type, *TSGH69Δwzy* K-antigen-deficient mutant, *TSGH69ΔwbbO* O-antigen-deficient mutant

Extracted CPS and LPS of two wildtype strains and four mutants were separated by means of SDS–PAGE and analyzed (Fig. 1). Silver staining, which detects LPS but not CPS, revealed that STL43Δ*wbbO* and TSGH69Δ*wbbO* had lost their LPS. Western immunoblotting with K1 antiserum, containing anti-K1 and anti-O1 antibodies, showed that STL43Δ*wzy* lost its K1 antigen, and TSGH69Δ*wbbO* its O1 antigen. Blotting with K2 antiserum, consisting anti-K2 and anti-O1 antibodies, revealed that STL43Δ*wbbO* lost its O1 antigen, and TSGH69Δ*wzy* its K2 antigen (Fig. 1).

**Fig. 2:**
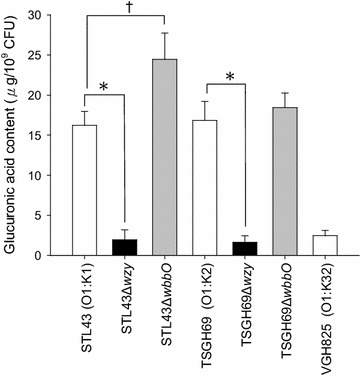
Glucuronic acid contents of seven *Klebsiella pneumoniae* strains. *STL43* serotype O1:K1 wild type, *STL43Δwzy* K-antigen-deficient mutant, *STL43ΔwbbO* O-antigen-deficient mutant. *TSGH69* serotype O1:K2 wild type, *TSGH69Δwzy* K-antigen-deficient mutant, *TSGH69ΔwbbO* O-antigen-deficient mutant. *VGH825* control strain. (*asterisk*) STL43 vs STL43Δ*wzy*, and (*asterisk*) TSGH69 vs TSGH69Δ*wzy* (for both, *P* < .001). (*dragger*) STL43 vs STL43Δ*wbbO* (*P* = .018). Chi square test statistical analysis was carried out for at least three successive tests in each isolate. The values are the mean ± standard deviation

Glucuronic acid, a component of the K1 and K2 antigen but not of the O1 and K32 antigen [19, 20], was measured to determine the amount of CPS. Glucuronic acid content of studied strains showed that STL43Δ*wzy* and TSGH69Δ*wzy* had lost their capsules, and STL43Δ*wbbO* and TSGH69Δ*wbbO* had retained their capsules (Fig. 2).

### Human neutrophil phagocytosis of wildtype and mutant strains

**Fig. 3:**
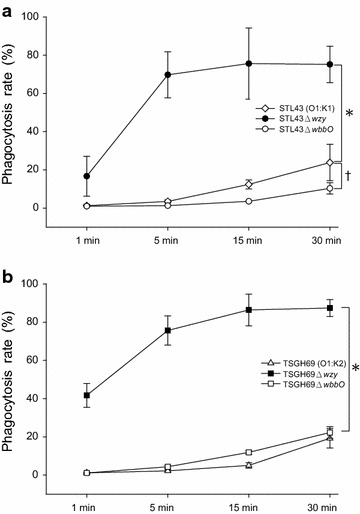
Phagocytosis rates of *Klebsiella pneumoniae* wildtype strains and their mutants. The percentage of neutrophils harboring fluorescein isothiocyanate (FITC)-stained bacteria was considered to be the phagocytosis rate. **a** *STL43* serotype O1:K1 wild type, *STL43Δwzy* K-antigen-deficient mutant, *STL43ΔwbbO* O-antigen-deficient mutant. (*asterisk*) STL43 vs STL43Δ*wzy* (*P* < .001). (*dragger*) STL43 vs STL43Δ*wbbO* (*P* = .006). **b** *TSGH69* serotype O1:K2 wild type, *TSGH69Δwzy* K-antigen-deficient mutant, *TSGH69ΔwbbO* O-antigen-deficient mutant. (*asterisk*) TSGH69 vs TSGH69Δ*wzy* (*P* < .001). Chi square test statistical analysis was carried out for at least six successive tests in each isolate. The values are the mean ± standard deviation

The contribution of K and O antigens to virulence was first evaluated with human neutrophil phagocytosis. Both of wildtype strains, STL43 and TSGH69, were resistant to phagocytosis (Fig. 3). While both of O deficient mutants, STL43Δ*wbbO* and TSGH69Δ*wbbO*, remained resistant to phagocytosis, K deficient mutants, STL43Δw*zy* and TSGH69Δ*wzy*, became susceptible to neutrophil phagocytosis. K antigen, either K1 or K2, has been showed as major determinant of phagocytosis resistance, and O antigen played no role in such resistance.

**Fig. 4:**
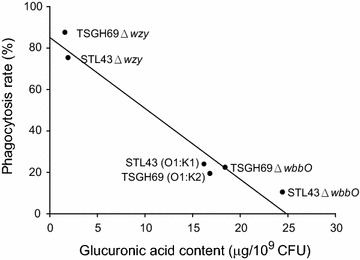
Association of phagocytosis rate and glucuronic acid contents of six *Klebsiella pneumoniae* strains. The percentage of neutrophils harboring fluorescein isothiocyanate (FITC)-stained bacteria after 30 min of incubation in six successive results was considered to be the phagocytosis rate. *STL43* serotype O1:K1 wild type, *STL43Δwzy* K-antigen-deficient mutant, *STL43ΔwbbO* O-antigen-deficient mutant, *TSGH69* serotype O1:K2 wild type, *TSGH69Δwzy* K-antigen-deficient mutant, *TSGH69ΔwbbO* O-antigen-deficient mutant

The association of glucuronic acid content and phagocytosis rate was analyzed (Fig. 4). Two K deficient mutants, TSGH69Δ*wzy* and STL43Δ*wzy*, had little amount of glucuronic acid, and were less resistant to phagocytosis. Two wildtype strain and two O deficient mutants had high glucuronic acid account, and were more phagocytosis resistant. The amount of glucuronic acid, indicating the amount of CPS, was inversed proportional to the rate of phagocytosis.

### Serum bactericidal assay of wildtype and mutant strains

**Fig. 5:**
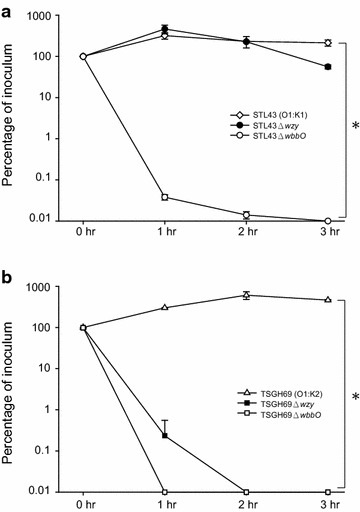
Serum bactericidal assay of *Klebsiella pneumoniae* wildtype strains and their mutants. **a** *STL43* serotype O1:K1 wild type, *STL43Δwzy* K-antigen-deficient mutant, *STL43ΔwbbO* O-antigen-deficient mutant. (*asterisk*) STL43 vs STL43Δ*wbbO* (*P* = .004). **b** *TSGH69* serotype O1:K2 wild type, *TSGH69Δwzy* K-antigen-deficient mutant, *TSGH69ΔwbbO* O-antigen-deficient mutant. (*asterisk*) TSGH69 vs TSGH69Δ*wzy*, and TSGH69 vs TSGH69Δ*wbbo* (for both, *P* = .006). Chi square test statistical analysis was carried out for at least two successive tests in each isolate. The values are the mean ± standard deviation

The virulence of six strains was evaluated with their resistance to human serum (Fig. 5). Both of wildtype strains were resistant to serum, with bacterial multiplication in human serum after 3 h. O deficient mutants, STL43Δ*wbbO* and TSGH69Δ*wbbO*, lost their wildtype serum resistance. While K2 deficient mutant, TSGH69Δ*wzy* was as susceptible to serum as its O deficient counterpart, K1 deficient STL43Δ*wzy* remained its wildtype serum resistance. O antigen was a major determinant in serum resistance in both serotype K1 and K2 strains. But the role of K antigen in serum resistance depended on its serotype.

### Hepatic bacterial clearance test of wildtype and mutant strains

The LD50 were less than 10 CFU for STL43 and TSGH69, about 3 × 10^5^ CFU for STL43Δ*wbbO* and TSGH69Δ*wbbO,* about 3 × 10^6^ CFU for STL43Δ*wzy* and 2 × 10^7^ CFU for TSGH69Δ*wzy.* Wildtype and mutant stains were peritoneally inoculated into mice at dose a tenth of LD_50_. Murine liver was harvested for bacterial clearance assay after 0, 3 h, and 1, 2, and 3 days.

**Fig. 6:**
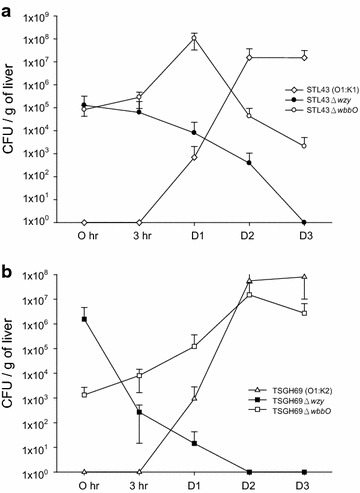
Liver bacterial clearance study of *Klebsiella pneumoniae* wildtype strains and their mutants. **a** *STL43* serotype O1:K1 wild type, *STL43Δwzy* K-antigen-deficient mutant, *STL43ΔwbbO* O-antigen-deficient mutant. **b** *TSGH69* serotype O1:K2 wild type, *TSGH69Δwzy* K-antigen-deficient mutant, *TSGH69ΔwbbO* O-antigen-deficient mutant. Chi square test statistical analysis was carried out for at least four successive tests in each isolate. The values are the mean ± standard deviation

In hepatic bacterial clearance assay, the wildtype strains, STL43 and TSGH69, proliferated in liver, and reached their plateaus at day 2 (Fig. 6). K deficient mutants, STL43Δ*wzy* and TSGH69Δ*wzy*, were eradicated within 3 days after inoculation. O deficient mutants, depending on its serotype, had different fate in liver. While STL43Δ*wbbO* diminished moderately at day 3, TSGH69Δ*wbbO* proliferated at the rate comparable to its wildtype counterpart.

### Liver histopathology of wildtype and mutant strains

**Fig. 7:**
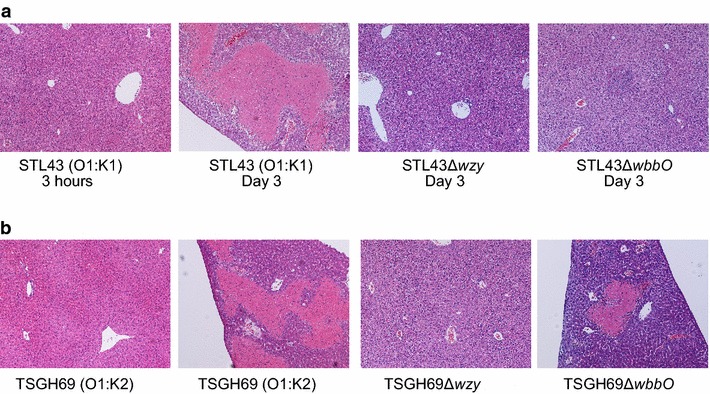
Histopathology of livers from C57BL/6 mice infected with *Klebsiella pneumoniae* strains. **a** *STL43 (O1:K1)* wild type, *STL43Δwzy* K-antigen-deficient mutant, *STL43ΔwbbO* O-antigen-deficient mutant. **b** *TSGH69 (O1:K2)* wild type, *TSGH69Δwzy* K-antigen-deficient mutant, *TSGH69ΔwbbO* O-antigen-deficient mutant

Fixed liver tissue was observed in a light microscope with magnitude of 200×. Wildtype strains, STL43 and TSGH59, caused profound necrosis and abscess formation in liver on day 3 (Fig. 7). K deficient mutants, STL43Δ*wzy* and TSGH69Δ*wzy*, caused no significant damage to liver. O deficient STL43Δ*wbbO* caused mild inflammation. But TSGH69Δ*wbbO* caused moderate necrosis, resembling its wildtype counterpart TSGH69.

## Discussion

Our work demonstrated the varied role of K and O antigens of serotype K1 and K2 *K. pneumoniae* in their virulence and the pathogenesis of liver abscess. The association between bacterial resistance to host immunity and histopathological finding of liver abscess was also elucidated.

While we have examined both of hepatic clearance and liver histopathology in several isolates before [21], this study further identified the association between bacterial resistance to hepatic clearance and its ability to cause pus formation. The strains susceptible to hepatic clearance, such as STL43Δ*wzy* and TSGH69Δ*wzy*, were unable to cause pus formation, while those resistant strains cause liver abscess. This indicated that bacterial resistance to hepatic bacterial clearance was compatible to the ability to cause pus formation, or liver abscess. This association may be quite straightforward, as more extended liver abscess contained more bacterial burden.

Two extrahepatic virulence factors, serum and neutrophil phagocytosis resistance, were analyzed for its predisposition to hepatic resistance in this study. Both of K1 and K2 wildtype *K. pneumoniae* strains were resistant to serum killing and hepatic clearance. But serum-resistant STL43Δ*wzy* was susceptible to hepatic clearance, and the serum-susceptible TSGH69Δ*wbbO* was resistant to hepatic clearance. Serum resistance was poorly correlated with hepatic resistance. Besides, our previous works showed liver abscess isolates were not significantly resistant to serum killing than stool carriage ones in serotype K1 (18/26 vs 11/21, P = 0.366) [22] and K2 (11/15 vs 7/11, P = 0.683) [23]. While the liver contains a lot of blood, serum plays a minor role in clearance of invaded *K. pneumoniae*.

But there was good correlation between resistance to phagocytosis and hepatic clearance, with phagocytosis-resistant strains resistant to hepatic clearance, vice versa. While phagocytosis rate seems to be a good predictor to hepatic clearance in some strains, STL43Δ*wbbO* was more resistant to phagocytosis than its wildtype, but was less resistant to hepatic clearance, and less able to cause pus formation. Besides, our previous study showed that not all of *K. pneumoniae* strains causing liver abscess were resistant to neutrophil phagocytosis [4]. The clearance of bacteria in liver attributes to fixed tissue macrophages, in particular to Kupffer cell [24]. The difference between phagocytosis of *K. pneumoniae* by neutrophils and Kupffer cells warrants further investigation.

The K and O antigens have long been regarded as factors in determining the resistance of *Klebsiella* [25, 26]. Those mutant strains which loss their K antigen loss their wildtype resistance to phagocytosis and liver clearance and ability to cause pus formation. The determinative role of K antigen in phagocytosis in serotype K1 and K2 strains was showed at this and previous studies [25]. But it is not always true to other serotypes. Loss of K7 antigen has been shown not affecting its wildtype phagocytosis [27]. However, glucuronic acid assay in this study indicated that the amount of CPS was associated with resistance to phagocytosis.

Previous studies have showed that serum resistance of *K. pneumoniae* was mediated by O antigen, and CPS played no role [28, 29]. But our study demonstrated the role of K antigen varied with its serotype, as K2 antigen was important in serum resistance, but K1 antigen was irrelevant.

While O antigen has been reported to contribute in the virulence [30], our study indicated that it was unrelated to neutrophil phagocytosis resistance, but a major factor of serum resistance. Though serotype K1 and K2 strains own the same O1 antigen, the O1 antigen, not of serotype K2, but of serotype K1, had some impact on liver clearance resistance and ability of pus formation.

The material to test resistance is an issue often been overlooked. Our study showed that O antigen is a major factor for human serum resistant, but O antigen has been reported not affecting mouse serum resistance [18]. Besides, normal human sera in one study contained sufficient concentrations of antibodies against *K. pneumoniae* [31]. The inconsistency between studies warrants further investigation.

## Conclusions

We conclude that the contribution of surface antigens to virulence of *K. pneumoniae* strains varies with serotypes. And it is an important concept to further virulence study about *K. pneumoniae.*

## References

[1] Podschun R, Ullmann U (1998). *Klebsiella* spp. as nosocomial pathogens: epidemiology, taxonomy, typing methods, and pathogenicity factors. Clin Microbiol Rev.

[2] Liu YC, Cheng DL, Lin CL (1986). Klebsiella pneumoniae liver abscess associated with septic endophthalmitis. Arch Intern Med.

[3] Lederman ER, Crum NF (2005). Pyogenic liver abscess with a focus on *Klebsiella pneumoniae* as a primary pathogen: an emerging disease with unique clinical characteristics. Am J Gastroenterol.

[4] Yeh KM, Kurup A, Siu LK, Koh YL, Fung CP, Lin JC (2007). Capsular serotype K1 or K2, rather than magA and rmpA, is a major virulence determinant for *Klebsiella pneumoniae* liver abscess in Singapore and Taiwan. J Clin Microbiol.

[5] Erbing C, Kenne L, Lindberg B, Lonngren J (1976). Structural studies of the capsular polysaccharide from *Klebsiella* Type 1. Carbohydr Res.

[6] Arakawa Y, Ohta M, Wacharotayankun R, Mori M, Kido N, Ito H (1991). Biosynthesis of *Klebsiella* K2 capsular polysaccharide in *Escherichia coli* HB101 requires the functions of rmpA and the chromosomal cps gene cluster of the virulent strain *Klebsiella pneumoniae* Chedid (O1:K2). Infect Immun.

[7] Whitfield C, Richards JC, Perry MB, Clarke BR, MacLean LL (1991). Expression of two structurally distinct d-galactan O antigens in the lipopolysaccharide of *Klebsiella pneumoniae* serotype O1. J Bacteriol.

[8] Vinogradov E, Frirdich E, MacLean LL, Perry MB, Petersen BO, Duus JO (2002). Structures of lipopolysaccharides from *Klebsiella pneumoniae*. Eluicidation of the structure of the linkage region between core and polysaccharide O chain and identification of the residues at the non-reducing termini of the O chains. J Biol Chem.

[9] Whitfield C, Roberts IS (1999). Structure, assembly and regulation of expression of capsules in *Escherichia coli*. Mol Microbiol.

[10] Chuang YP, Fang CT, Lai SY, Chang SC, Wang JT (2006). Genetic determinants of capsular serotype K1 of *Klebsiella pneumoniae* causing primary pyogenic liver abscess. J Infect Dis.

[11] Clarke BR, Whitfield C (1992). Molecular cloning of the rfb region of *Klebsiella pneumoniae* serotype O1:K20: the rfb gene cluster is responsible for synthesis of the d-galactan I O polysaccharide. J Bacteriol.

[12] Hansen DS, Mestre F, Alberti S, Hernandez-Alles S, Alvarez D, Domenech-Sanchez A (1999). *Klebsiella pneumoniae* lipopolysaccharide O typing: revision of prototype strains and O-group distribution among clinical isolates from different sources and countries. J Clin Microbiol.

[13] Yeh KM, Lin JC, Yin FY, Fung CP, Hung HC, Siu LK (2010). Revisiting the importance of virulence determinant magA and its surrounding genes in *Klebsiella pneumoniae* causing pyogenic liver abscesses: exact role in serotype K1 capsule formation. J Infect Dis.

[14] Ohtomo R, Saito M (2003). A new selective medium for detection of *Klebsiella* form diary environments. Microbes Environ.

[15] Tsai CM, Frasch CE (1982). A sensitive silver stain for detecting lipopolysaccharides in polyacrylamide gels. Anal Biochem.

[16] Domenico P, Schwartz S, Cunha BA (1989). Reduction of capsular polysaccharide production in *Klebsiella pneumoniae* by sodium salicylate. Infect Immun.

[17] Podschun R, Fischer A, Ullman U (2000). Expression of putative virulence factors by clinical isolates of *Klebsiella planticola*. J Med Microbiol.

[18] Shankar-Sinha S, Valencia GA, Janes BK, Rosenberg JK, Whitfield C, Bender RA (2004). The *Klebsiella pneumoniae* O antigen contributes to bacteremia and lethality during murine pneumonia. Infect Immun.

[19] Athamna A, Ofek I, Keisari Y, Markowitz S, Dutton GG, Sharon N (1991). Lectinophagocytosis of encapsulated *Klebsiella pneumoniae* mediated by surface lectins of guinea pig alveolar macrophages and human monocyte-derived macrophages. Infect Immun.

[20] Guan S, Clarke AJ, Whitfield C (2001). Functional analysis of the galactosyltransferases required for biosynthesis of d-galactan I, a component of the lipopolysaccharide O1 antigen of *Klebsiella pneumoniae*. J Bacteriol.

[21] Fung CP, Chang FY, Lin JC, Ho DM, Chen CT, Chen JH (2011). Immune response and pathophysiological features of *Klebsiella pneumoniae* liver abscesses in an animal model. Lab Invest J Tech Methods Pathol.

[22] Siu LK, Fung CP, Chang FY, Lee N, Yeh KM, Koh TH (2011). Molecular typing and virulence analysis of serotype K1 *Klebsiella pneumoniae* strains isolated from liver abscess patients and stool samples from noninfectious subjects in Hong Kong, Singapore, and Taiwan. J Clin Microbiol.

[23] Lin JC, Koh TH, Lee N, Fung CP, Chang FY, Tsai YK (2014). Genotypes and virulence in serotype K2 *Klebsiella pneumoniae* from liver abscess and non-infectious carriers in Hong Kong, Singapore and Taiwan. Gut Pathog.

[24] Bilzer M, Roggel F, Gerbes AL (2006). Role of Kupffer cells in host defense and liver disease. Liver Int Off J Int Assoc Study Liver.

[25] Simoons-Smit AM, Verweij-van Vught AM, MacLaren DM (1986). The role of K antigens as virulence factors in *Klebsiella*. J Med Microbiol.

[26] Williams P, Lambert PA, Brown MR, Jones RJ (1983). The role of the O and K antigens in determining the resistance of *Klebsiella aerogenes* to serum killing and phagocytosis. J Gen Microbiol.

[27] Podschun R, Penner I, Ullmann U (1992). Interaction of *Klebsiella* capsule type 7 with human polymorphonuclear leucocytes. Microb Pathog.

[28] Tomas JM, Benedi VJ, Ciurana B, Jofre J (1986). Role of capsule and O antigen in resistance of *Klebsiella pneumoniae* to serum bactericidal activity. Infect Immun.

[29] Ciurana B, Tomas JM (1987). Role of lipopolysaccharide and complement in susceptibility of *Klebsiella pneumoniae* to nonimmune serum. Infect Immun.

[30] Hsieh PF, Lin TL, Yang FL, Wu MC, Pan YJ, Wu SH (2012). Lipopolysaccharide O1 antigen contributes to the virulence in *Klebsiella pneumoniae* causing pyogenic liver abscess. PLoS One.

[31] Lepper PM, Moricke A, Held TK, Schneider EM, Trautmann M (2003). K-antigen-specific, but not O-antigen-specific natural human serum antibodies promote phagocytosis of Klebsiella pneumoniae. FEMS Immunol Med Microbiol.

